# *De-novo* synthesis of 2-phenylethanol by *Enterobacter* sp. CGMCC 5087

**DOI:** 10.1186/1472-6750-14-30

**Published:** 2014-04-25

**Authors:** Haibo Zhang, Mingle Cao, Xinglin Jiang, Huibin Zou, Cong Wang, Xin Xu, Mo Xian

**Affiliations:** 1 CAS Key Laboratory of Biobased Materials, Qingdao Institute of Bioenergy and Bioprocess Technology, Chinese Academy of Sciences, No.189 Songling Road, Laoshan District, Qingdao, PR 266101, China; 2 University of Marburg, Marburg, Germany

**Keywords:** 2-Phenylethanol, Biosynthesis, Ehrlich pathway, *Enterobacter* sp

## Abstract

**Background:**

2-phenylethanl (2-PE) and its derivatives are important chemicals, which are widely used in food materials and fine chemical industries and polymers and it’s also a potentially valuable alcohol for next-generation biofuel. However, the biosynthesis of 2-PE are mainly biotransformed from phenylalanine, the price of which barred the production. Therefore, it is necessary to seek more sustainable technologies for 2-PE production.

**Results:**

A new strain which produces 2-PE through the phenylpyruvate pathway was isolated and identified as *Enterobacter* sp. CGMCC 5087. The strain is able to use renewable monosaccharide as the carbon source and NH_4_Cl as the nitrogen source to produce 2-PE. Two genes of rate-limiting enzymes, chorismate mutase *p*-prephenate dehydratase (PheA) and 3-deoxy-*d*-arabino-heptulosonic acid 7-phosphate synthase (DAHP), were cloned from *Escherichia coli* and overexpressed in *E.* sp. CGMCC 5087. The engineered *E.* sp. CGMCC 5087 produces 334.9 mg L^-1^ 2-PE in 12 h, which is 3.26 times as high as the wild strain.

**Conclusions:**

The phenylpyruvate pathway and the substrate specificity of 2-keto-acid decarboxylase towards phenylpyruvate were found in *E.* sp. CGMCC 5087. Combined with the low-cost monosaccharide as the substrate, the finding provides a novel and potential way for 2-PE production.

## Background

2-Phenylethanol (2-PE), an aromatic alcohol with rose-like odour, is widely used in the food, drink and cosmetic industry, and it’s also a potentially valuable alcohol for next-generation biofuel [1]. Additionally, 2-PE is an important raw material for its derivatives, among which phenylethyl acetate is a valuable fragrance compound, and *p*-hydroxyphenylethanol is widely used in pharmaceutical and fine chemical industries [2,3].

2-PE can be synthesized by chemical and biological methods. Up to now, the vast majority of 2-PE is produced chemically, while purification of the product is still a key problem in these chemical processes. The quality of the chemical synthesized 2-PE is reduced greatly because of its harsh conditions and the toxic reagents. 2-PE naturally exists in rose flower, and a number of fungi are known as 2-PE producers including *Phellinus ignarius*, *Ischnoderma benzoinum*, *Geotrichum penicillatum*, *Kluyveromyces marxianus*, *Saccharomyces cerevisiae*, *Aspergillus niger* etc. [4]–[7]. *S. cerevisiae* could produce 4.5 g L^-1^ 2-PE in fed-batch fermentation [4]. When oleic acid was used as the organic phase for two-phase fed-batch cultures, *S. cerevisiae* could produce 12.6 g L^-1^ 2-PE [8]. However, all these fermentations use phenylalanine as feedstock, the prices of which barred the industrial scale production of 2-PE. Very few bacteria were found to synthesize 2-PE. When using aromatic amino acids as nitrogen source, *Erwinia carotovora* was proved to produce 2-PE [9]. Jollivet *et al.* found 2-PE in the culture of *Microbacterium* sp. and *Brevibacterium linens*, while no further progress was reported [10].

**Figure 1:**
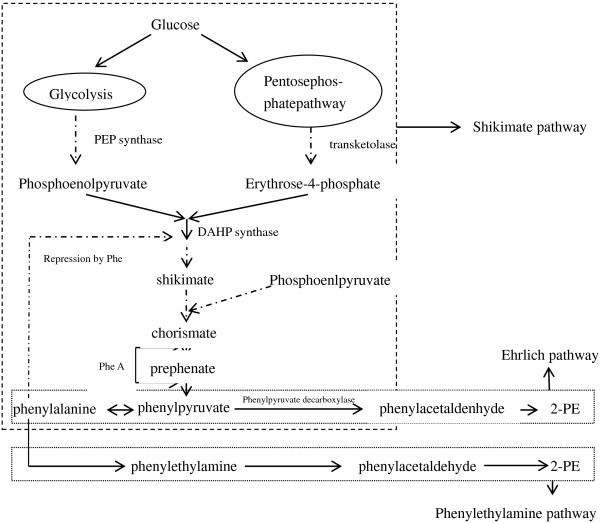
**Phenylpyruvate pathway for 2-PE synthesis.** 2-PE can be biosynthesized from phenylalanine through the Phenylethylamine pathway and the Ehrlich pathway. The shikimate pathway and the Ehrlich pathway form a phenylpyruvate pathway, which can *de-novo* synthesize 2-PE from glucose.

2-PE can be biosynthesized from phenylalanine through the Phenylethylamine pathway and the Ehrlich pathway in eukaryotes (Figure 1, http://www.kegg.jp/kegg-bin/highlight_pathway?scale=1.0&map=map00360&keyword=phenethyl alcohol), and the latter one is thought to be the most significant pathway [2]. The shikimate pathway and the Ehrlich pathway can form a phenylpyruvate pathway, which can *de-novo* synthesize 2-PE from glucose. Hwang J-Y *et al.* constructed the yeast Ehrlich pathway into *E. coli*, but the failure of overexpressing all the foreign proteins in soluble and active form blocked the high production of 2-PE [11], and they also used phenylalanine as feedstock. James C. Liao *et al.* engineered *E. coli* to produce varied alcohols by overexpressing different heterologous 2-keto-acid decarboxylases (KDCs) and alcohol dehydrogenases, and 2-PE was detected among these alcohols, but the substrate specificity of KDCs was broad and no further research was performed for 2-PE [12]. Whether increasing carbon flux to the 2-keto acids could improve the productivity of the alcohol or not is still needed to be verified.

In our study, a new strain *Enterobacter* sp. CGMCC 5087 was isolated and identified. The strain was verified to produce 2-PE using *de-novo* synthetic pathway with monosaccharide as a carbon source and NH_4_Cl as a nitrogen source. Two rate-limiting enzymes, PheA and DAHP synthase, were overexpressed to increase the flux to 2-keto phenylpyruvate to further increase production of 2-PE.

## Results and discussion

### Identification of the strain

The bacterium was accidentally obtained when we screened strains against 5 g L^-1^ resorcinol, and 2-PE was detected when it was cultured in the selective medium. The screened strain is rod-shaped, with width from 0.3 to 0.5 μm and length from 1 to 2 μm. And it is an aerobic bacterium. 16S rDNA gene sequence (1412 bp) was blasted at NCBI against Genome Survey Sequence (GSS). Based on these results, this strain was identified to be *E*. sp. and preserved in China General Microbiological Culture Collection Center (CGMCC) as *E*. sp. CGMCC 5087. 2-PE has been used as a bactericide in pharmaceutical preparations for many years [13]. 2-PE was thought to induce the breakdown of the cell membrane limitedly and inhibit the synthesis of deoxyribonucleic acid [14]. Secretion of 2-PE by this bacteria may be a bactericidal substance for interspecies competition.

Many microorganisms, especially yeasts, have been reported to produce 2-PE by normal metabolism, however, the final concentration of 2-PE in the culture broth of selected strains remains very low. That was why all the fermentation for 2-PE used phenylalanine as nitrogen source. Besides, very few wild prokaryotic organisms were reported to secrete 2-PE. Therefore, validation of 2-PE *de-novo* biosynthesis pathway is not only useful metabolic engineering but also valuable for theoretical research.

### Validating of 2-PE *de-novo* biosynthesis in *E*. sp. CGMCC 5087

**Figure 2:**
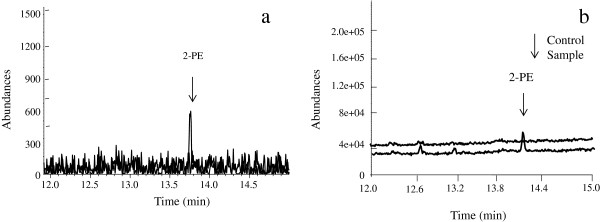
**Validating of biosynthesis of 2-PE by *E. *sp. CGMCC 5087. a**. *E.* sp. CGMCC 5087 was cultured in M9 medium and detected with GC-MS; **b**. *E.* sp. CGMCC 5087 was cultured in LB medium and detected with GC.

As the selective medium contains beef and yeast extract, M9 medium was used to validate whether *E*. sp. CGMCC 5087 can biosynthesize 2-PE using glucose as the sole carbon source. After *E.* sp. CGMCC 5087 was grown in M9 medium at 37°C for 24 h, 2-PE was detected. Characteristic ion (m/z = 122) spectra was shown in Figure 2b. The production of 2-PE in M9 medium was much lower (Figure. 2b) than in LB medium (70 mg L^-1^, Figure 2a).

The results indicated that *E*. sp. CGMCC 5087 can synthesize 2-PE using *de-novo* pathway with monosaccharide as carbon source and NH_4_Cl as nitrogen source. To the best of our knowledge, this is the first wild bacterium validated to produce 2-PE using glucose as sole carbon source. Although phenylalanine is mostly used as fermentation stock for 2-PE biosynthesis, the high price is still a huge disadvantage. The cheaper glucose makes this strain more competitive for industrial production.

Most microorganisms can synthesize phenylalanine via shikimate pathway, and subsequently phenylalanine can be converted to 2-PE by many microorganisms through Phenylethylamine pathway or Ehrlich pathway [2]. The shikimate pathway and the Ehrlich pathway are connected by the phenylpyruvate to form a phenylpyruvate pathway, which can *de-novo* synthesize 2-PE from glucose. Validating the synthetic pathway of 2-PE would be helpful for the further research.

### Validating phenylpyruvate pathway in *E*. sp. CGMCC 5087

**Figure 3:**
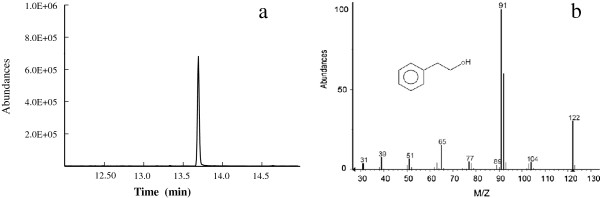
**Validating Ehrlich pathway in *E. *sp. CGMCC 5087. a**. GC chromatography of 2-PE biocatalyzed with crude enzymes with phenylpyruvate as substrate; **b**. MS spectrum of 2-PE biocatalyzed with crude enzymes with phenylpyruvate as substrate.

We examined the activity of crude enzymes to identify 2-PE biosynthesis pathway involved in *E.* sp. CGMCC 5087. The results indicated that crude enzymes can catalyze phenylpyruvate to 2-PE (Figure 3). As phenylpyruvate is not the intermediate product of Phenylethylamine pathway, the occurrence of 2-PE suggested that it is biotransformed from phenylpyruvate through Ehrlich pathway, which is consistent with the pathway that James C. liao and Hwang J-Y constructed [11,12].

Meanwhile, the results indicate that 2-keto-acid decarboxylase (KDC) is produced by *E.* sp. CGMCC 5087. KDCs have a broad substrate range and widely exist in plants, yeasts and fungi, but less so in bacteria [15]. No other alcohols such as 1-butanol were detected (data not shown) indicates that the KDC has a good substrate specificity [12]. In the Ehrlich pathway, phenylpyruvate was converted to phenylacetaldehyde by KDC firstly, and then converted to 2-PE by alcohol dehydrogenase (ADH) with nicotinamide adenine dinucleotide phosphate (NADPH) as reducing agent. As importing and expressing non-native pathways may lead to the metabolic imbalance, and also heterologous metabolites may exhibit cytotoxicity [3,16,17], co-existence of phenylpyruvate pathway and the two enzymes KDC and ADH makes *E.* sp. CGMCC 5087 a good candidate to produce 2-PE.

### Biosynthesis of 2-PE with engineered *E*. sp. CGMCC 5087

The existing shikimate pathway was then genetically modified to increase 2-PE production by enhancing the formation of phenylpyruvate. Both chorismate mutase *p*-prephenate dehydratase (PheA) and 3-deoxy-*d*-arabino-heptulosonic acid 7-phosphate synthase (DAHP synthase) are proved to be rate-limiting enzymes in the L-phenylalanine synthesis pathway. Therefore, enhancing the expression level of these two enzymes will increase the flux to 2-keto-phenylbutyric acid. As is shown in Figure 1, Carbon flow through the shikimate pathway is controlled by modulation of DAHP. In *E. coli*, three unlinked genes *aro*F, *aro*G and *aro*H encode three isoenzymes of DAHP synthase, respectively. Among these genes, *aro*F was proved to play a positive effect for biosynthesis of phenylalanine [18,19]. Chorismate mutase *p*-prephenate dehydratase was also identified as a rate-limiting enzyme for the synthesis of phenylalanine [14,20]. pMW13, an expression vector identified to be able to replicate in *E.* sp. CGMCC 5087 was used to express pheA and aroF. Expression of the proteins was evaluated by SDS-PAGE analysis. Two specific banDs, with the right molecular masses, were able to be observed apparently at the recombinant strain, compared to the negative control (data not shown) [21].

**Figure 4:**
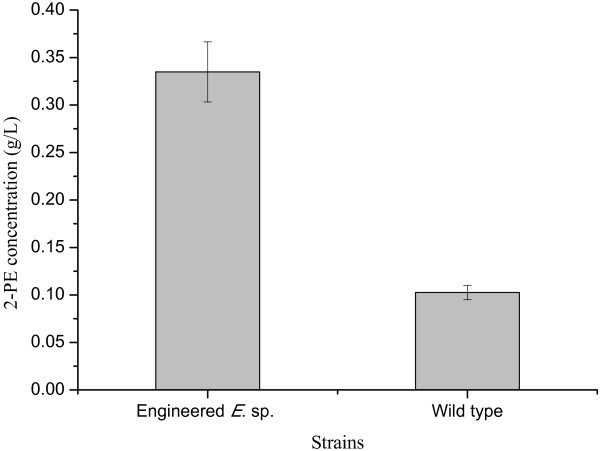
**The production of 2-PE by the wild and engineered *E. *sp. CGMCC 5087.** Control: wild type *E.* sp. CGMCC 5087; Engineered *E.* sp. CGMCC 5087: *E.* sp. CGMCC 5087 harboring pMW13. The wild type *E.* sp. CGMCC 5087 and the engineered strain can produce 102.6 and 334.9 mg L^-1^ 2-PE, respectively. All the experiments were performed in triplicate, and error bars indicate standard deviations.

The wild type *E.* sp. CGMCC 5087 can produce 102.6 mg L^-1^ 2-PE when cultured in production medium for about 12 h (Figure 4). The plasmid pMW13_*phe*A_*aro*F was transformed to enhance phenylpyruvate biosynthesis to combine with the alcohol producing pathway to achieve 2-PE production. The 2-PE production was about 334.9 mg L^-1^after incubation in shake flask for 12 h, and it is 3.26 times as high as the wild strain. When phenylalanine was added to the production medium at the concentration of 5 g L^-1^, 2-PE production was 530 mg L^-1^. It’s much higher than the 469 μM (about 57 mg L^-1^) that James C. Liao *et al*. reported [12].

According to phenylpyruvate pathway, two mol phosphoenolpyruvate, one mol erythrose-4-phosphate and one mol adenosine triphosphate can form one mol phenylpyruvate which can be then converted to one mol 2-PE with two mol NADPH then. According to the maximum theoretical yield, when 1 g 2-PE was produced, 2.53 g glucose or 1.34 g phenylalanine was needed. However, the price of phenylalanine (55, 000 ¥/ton) was about 17 folds that of glucose (3, 200 ¥/ton) in China. The cheaper sugars make the strain more competitive for industrial production. Though the titer of 334.9 mg L^-1^ was still lower than the previous report by *S. cerevisiae*, further optimization, including fed-batch fermentation, addition of extractive solvent, could be done to elevate the yield of 2-PE [4,8].

## Conclusions

A new strain *E.* sp. CGMCC 5087, which is able to produce 2-PE using *de-novo* pathway with monosaccharide as carbon source and NH_4_Cl as nitrogen source was isolated and identified. The strain can synthesize 2-PE through phenylpyruvate pathway. Two rate-limiting enzymes, PheA and DAHP synthase from *E. coli* were then overexpressed in *E.* sp. CGMCC 5087 to construct engineered *E.* sp for 2-PE production. The engineered strain can produce 334.9 mg L^-1^ 2-PE for about 12 h. The substrate specificity of 2-keto-acid decarboxylase and the existence of phenylpyruvate pathway make *E.* sp. CGMCC 5087 a good candidate for 2-PE production.

## Methods

### Chemicals

Phenylpyruvate, phenylethanol and 2-ethylbutyric acid were purchased from Sigma-Aldrich (China). NADPH was obtained from Roche Products (China). The Bacterial DNA Kit and the Plasmid Mega Kit were bought from OMEGA. The Competent Cell Preparation Kit was from TaKaRa (Dalian, China). The other reagents were all analytical grades.

### Strains

*E.* sp. CGMCC 5087 was isolated from the sewage sludge of dyes wastewater with a selective medium. The sewage sludge was diluted with sterile water and incubated on the selective medium with 1.2% agar. Grown clones were then incubated into 50 ml of LB medium, cultured for 24 h, and extracted with ethyl acetate. Extractions were identified with gas chromatography–mass spectrometry as mentioned below. The genomic DNA of the isolated strain was extracted using Bacterial DNA Kit. 16S rDNA was amplified using the consensus primers fD1 (5′-AGAGTTTGATCCTGGCTCAG-3′) and rP2 (5′-CGGCTACCTTGTTACGACTT-3′), and PCR products were cloned into pMD™ 18-T vector. Clones derived from the PCR products were confirmed by restriction analyses and sequenced. *Escherichia coli* MWPEC13-60(ATCC 67459) with plasmid pMW13_*phe*A_*aro*F was purchased from the American Type Culture Collection [22]. The strains and plasmids are shown in Table 1.

**Table 1:** Bacterial strains and plasmids used in this study

**Plasmids or strains**	**Relevant genotype or description**	**Reference**
Plasmids		
pMDTM18-T	pUC origin; Amp^R^	Takara
pMW13	pMW origin; Kana^R^; P_PL-PR_::*pheA*, *aroF*	[20]
Strains		
DH5α	deoR, recA1, endA1, hsdR17(rk^-^, mk^+^), phoA, supE44, λ^-^, thi^-^1, gyrA96, relA1	Takara
*E*. sp.	*Enterobacter* sp. CGMCC 5087 Amp^R^	This study
*E.coli* MWPEC13-60	*E. coli* harboring pMW13	ATCC® 67459™
Engineered *Ec* sp.	*E*. sp. harboring pMW13	This study

### Medium

The composition of the selective medium contains: resorcinol, 5 g L^-1^; glucose, 5 g L^-1^; beef extract, 2 g L^-1^; tryptone, 7 g L^-1^; NaC1, 4 g L^-1^; pH 7.0 ~ 7.4. With glucose as carbon source and NH_4_Cl as nitrogen source, M9 medium (NH_4_Cl, 1 g L^-1^; Na_2_HPO_4_ · 7H_2_O, 11 g L^-1^; KH_2_PO_4_, 3 g L^-1^; NaC1, 0.5 g L^-1^; glucose, 20 g L^-1^; MgSO_4_, 120 mg L^-1^; CaC1_2_, 10 mg L^-1^; pH 7.0 ~ 7.4) was used to verify *de-novo* biosynthesis of 2-PE by *E.* sp. CGMCC 5087. Luria-Bertani (LB) medium (tryptone 1%, yeast extract 0.5%, sodium chloride 1%, pH 7.0 ~ 7.4) was used to culture *E.* sp. CGMCC 5087 and *E. coli*. production medium for 2-PE contains the following components: sucrose, 20 g L^-1^; yeast extraction, 5 g L^-1^; KH_2_PO_4_, 3 g L^-1^; K_2_HPO_4_; 3.82 g L^-1^; MgSO_4_ · 7H_2_O, 0.5 g L^-1^; Na_2_MoO_4_, 0.01 g L^-1^; CaCl_2_, 0.5 g L^-1^; NaCl, 1.0 g L^-1^, pH 7.2 ~ 7.5. LB mediums with ampicillin (50 μg ml^-1^) and kanamycin (50 μg ml^-1^) were used to culture the strains with pMD™ 18-T vector and pMW13_*phe*A_*aro*F, respectively.

### Analytical methods

2-PE was quantified by gas chromatography (GC), which has a FID and HP Innowax capillary column (30 m × 0.25 mm, 0.25 μm film thickness). The chromatographic conditions were: column temperature 50°C (1 min), 50–240°C (15°C/min), 240°C (15 min), injector temperature 250°C, detector temperature 280°C, carrier gas nitrogen (0.2 kPa), flow rate 1.0 ml min^-1^, volume injected 0.4 μl. 2-Ethylbutyric acid was used as interior standard. 2-PE was identified with gas chromatography–mass spectrometry (GC-MS; Agilent 6890 N GC and 5975 inert MSD) using a pulsed pressure injection of 1 μl onto an Innowax-101025 column (30 m × 0.25 mm, 0.25 μm film thickness), ion source (EI) ionization, and a dwell time of 40 msec were applied for each characteristic ion. The GC-MS chromatographic conditions were the same as GC conditions mentioned above.

### Verifying *de-novo* biosynthesis of 2-PE by *E*. sp. CGMCC 5087

*E.* sp. CGMCC 5087 was inoculated to M9 medium (50 ml) and incubated at 37°C on a shaker (150 rpm) for 24 h. The culture was then centrifuged at 12, 000 rpm for 1 min, and the supernatant with a volume of 45 ml was extracted with 5 ml of n-butyl alcohol. Extraction was filtrated with 0.22 μm polyether sulphone membrane and qualitatively analyzed the 2-PE content with GC-MS. M9 medium without any inoculation and dealt with the same conditions was used as control.

### Verifying phenylpyruvate pathway in *E*. sp. CGMCC 5087

As the key intermediate of the phenylpyruvate pathway, phenylpyruvate was added to verify the pathway in *E.* sp. CGMCC 5087. The strain was inoculated to LB medium and incubated at 37°C on a shaker (150 rpm) for 12 h. The culture was centrifuged at 12, 000 rpm for 2 min. Deposition cell was washed and suspended with 50 mM phosphate buffers (pH 7.2). The cells were lysed by ultrasonication in the ice bath. The lysed solution was centrifuged at 12,000 rpm for 1 min and supernatant was used as crude enzyme to catalyze the conversion of phenylpyruvate to 2-PE with1 μM NADPH. The reaction was incubated at 37°C for 2 h and ended by heating up at 75°C for 30 min. The control was made by heating the crude enzymes and NADPH mixture before the substrate was added. The mixtures were centrifuged at 12, 000 rpm for 20 min, and the supernatant were detected with GC-MS for the 2-PE contents.

### Plasmid and gene expression

Plasmid pMW13_*pheA*_*aroF* was constructed by Lee *et al.*[22], which contains the *pheA* (GI: 254547524), and *aroF* (GI: 145363), under a temperature sensitive promoter (PL-PR promoter of the lambda phage). *AroF* and *pheA* encode DAHP synthase and PheA, respectively. *E.* sp. CGMCC 5087 competent cell was prepare by the Competent Cell Preparation Kit (TaKaRa, Dalian, China). The plasmid pMW13_*pheA_aroF* was transformed into the competent cells, and the positive transformants were screened against kanamycin (50 μM). The engineered *E.* sp. CGMCC 5087 was incubated at 32°C to overexpress proteins.

### Biosynthesis of 2-PE with engineered *E*. sp. CGMCC 5087

The engineered *E.* sp. CGMCC 5087 was inoculated into production medium and incubated at 32°C on a shaker (150 rpm) for 12 h. The culture was centrifuged at 12, 000 rpm for 2 min, and the filtered supernatant was quantified its 2-PE content with GC. Wild type *E.* sp. CGMCC 5087 was cultured and measured its 2PE content as the control. Wild type *E.* sp. CGMCC 5087 was cultured in the production medium with 5 g L^-1^ phenylalanine as another control to verify the effects of phenylalanine.

### Calculations

The maximum theoretical yield is calculated by assuming that the branching pathways are blocked and that the carbon flow is directed by the most efficient pathways with minimum loss to carbon dioxide and other metabolites [23,24]. At the theoretical level, 1 mol of 2-PE and 4 mol of NADPH can be produced from 2 mol of glucose and 2 mol of ATP.

The following equation was used to calculate the maximum theoretical yield.

$$Q_{p}=\frac{N_{p}M_{p}}{N_{g}M_{g}}\times 100\%$$

where Q_p_ = the maximum theoretical yield; N = the number of moles (mol); M = molecular weight; p = 2-PE; g = glucose.

## Abbreviations

ADH: Alcohol dehydrogenase; CGMCC: China general microbiological culture collection center; DAHP: 3-deoxy-*d*-arabino-heptulosonic acid 7-phosphate synthase; FID: Flame ionization detector; GC: Gas chromatography; GC-MS: Gas chromatography-mass spectrum; GSS: Genome Survey Sequence; 2-PE: 2-phenylethanl; KDC: 2-keto-acid decarboxylase; KDCs: 2-keto-acid decarboxylases; NADPH: Nicotinamide adenine dinucleotide phosphate; PCR: Polymerase chain reaction; PheA: Chorismate mutase *p*-prephenate dehydratase.

## Competing interests

The authors declare that they have no competing interests.

## Authors’ contributions

MX developed the idea for the study. HZh designed the research, did the literature review and prepared the manuscript. MX helped to revise the manuscript. MC, XJ, HZo, CW and XX did the lab work, plasmid construction, strain cultivation and product detection. All authors read and approved the final manuscript.
